# The Effect of Heat Input, Annealing, and Deformation Treatment on Structure and Mechanical Properties of Electron Beam Additive Manufactured (EBAM) Silicon Bronze

**DOI:** 10.3390/ma15093209

**Published:** 2022-04-29

**Authors:** Andrey Filippov, Nikolay Shamarin, Evgeny Moskvichev, Nikolai Savchenko, Evgeny Kolubaev, Ekaterina Khoroshko, Sergei Tarasov

**Affiliations:** Institute of Strength Physics and Materials Science, Siberian Branch of the Russian Academy of Sciences, Pr. Akademicheskiy 2/4, 634055 Tomsk, Russia; shamarin.nik@gmail.com (N.S.); em_tsu@mail.ru (E.M.); savnick@ispms.tsc.ru (N.S.); eak@ispms.ru (E.K.); eskhoroshko@gmail.com (E.K.); tsy@ispms.ru (S.T.)

**Keywords:** additive manufacturing, heat input, silicon bronze, microstructure, zigzagged columnar grains, tensile strength, hardness

## Abstract

Electron beam additive wire-feed manufacturing of Cu-3wt.%S-0.8wt.%Mn bronze thin wall on a stainless steel substrate has been carried out at heat input levels of 0.19, 0.25, and 0.31 kJ/mm. The microstructures of as-deposited metal ranged from low aspect ratio columnar with equiaxed grain layers to zig-zagged and high aspect ratio columnar, as depended on the heat input. Post-deposition annealing at 900 °C for 6 h resulted in recrystallization of the high aspect ratio columnar grains with further grain growth by boundary migration. Pre-deformation by 10% thickness reduction and then annealing at 900 °C for 6 h also allowed obtaining recrystallized grain structures with less fraction of twin boundaries but higher fraction of high-angle ones, as compared to those of only annealed sample. Pre-deformation and ensuing annealing allowed simultaneous increasing of the ultimate tensile strength and strain-to-fracture.

## 1. Introduction

Copper alloys are widely used in many industrial processes as heat and electro-conductive as well as tribotechnical materials. Among them, silicon copper alloys are applied for fabricating components that should combine very high levels of mechanical characteristics with good processability and high corrosion and wear resistance, for example, fittings, journal bearings, vessels, etc.

Traditional production of copper alloy components is undertaken by casting with the ensuing mechanical processing [1,2]. As-cast bronze microstructures are usually represented by large columnar grains that are detrimental for mechanical characteristics, and therefore various thermomechanical post-treatments such as forging and rolling should be used to improve them. However, it is very difficult to apply these treatments for processing either large-sized or shaped additively manufactured components [3,4,5,6,7].

Another approach is feasible that allows modifying only subsurface layer structures by irradiation the targets with concentrated energy sources, such as electron beam, laser, and arc discharge. For instance, arc surfacing was used to improve hardness and wear resistance of silicon copper alloy by grain refining [8]. Laser surfacing on C63200 bronze resulted in reducing ductility and increasing the yield strength, while keeping the ultimate strength constant [9]. These surface treatments cannot indeed provide grain structure modification in the bulk of the component, which is not always acceptable from the viewpoint of loading.

Nowadays, there is a fast development of additive manufacturing that allows obtaining near net shape components, which require only minimal post-processing. Another advantage is a potential for fabricating shaped components with internal cavities that are very hard to obtain by subtractive metal processing.

Research literature sources [10,11,12,13] show that microstructures of the additively manufactured components may be similar with those obtained by casting, i.e., there may be coarse dendrites or columnar grains that impair mechanical and even functional characteristics of the manufactured components. For example, samples obtained using electron beam additive manufacturing (EBAM) from copper wire [14] and wire-arc additive manufacturing (WAAM) from a silicon bronze wire [15] were characterized by coarse columnar grain structures and corresponding anisotropy of the mechanical characteristics. The ultimate tensile strength varied from 145 to 394 MPa depending upon the sampling zone. The grain size was changing from ~9 μm to ~1 mm.

Solutions to improving the columnar grain structures lie either with post-processing or with adjusting the additive process parameters in order to control solidification of the melted pool, and thus improve the grain structure uniformity.

Heat input is the most important parameter to control during additive manufacturing (AM) on various metals and alloys. Such an approach was applied by a number of researchers during additive manufacturing on alloys inclined to forming large columnar grains, namely nickel superalloys [16], Ti-6Al-4V alloy [17], copper [18], Cu-Al-Si-Mn bronze [19], NiTi shape memory alloy [20,21], etc. Nevertheless, despite improving the grain structure, the use of only heat input control did not necessarily mean improving the mechanical characteristics. It was shown [22] that despite some uniform grain refining effect being achieved due to the heat input control, the mechanical strength of the as-deposited thin-walled Cu-7wt.%Al bronze could be higher.

More encouraging results were obtained on post-processing the as-deposited thin-walled Cu-7wt.%Al bronze with the use of heat treatment and deformation [23]. The recrystallized grains were almost equiaxed with numerous twin boundaries, which served for simultaneous improvement of both ultimate strength and plasticity. Similar results were obtained elsewhere [24].

It was shown earlier [25,26,27] that additive manufacturing on silicon aluminum bronze (SAB) also resulted in forming non-homogeneous grain structures, as viewed from the bottom to the top of the wall. Therefore, each of the above-described approaches can be used for improving the characteristics of the additively manufactured samples. The cold-metal transfer fabricated SAB was subjected to cryogenic treatment that not only allowed forming fine-grained structures with high amount of high-angle grain boundaries, but also improved the ultimate tensile strength by 20%, as compared to that of the as-deposited SAB [26]. Laser metal deposition (LMD) additive manufacturing was used to fabricate a Cu-9Al-5Fe-5Ni bronze, where structures and mechanical characteristics of the additive samples were controlled by changing the heat input level [28]. WAAM fabricated nickel aluminum bronze (NAB) was subjected to annealing at 350 °C for 2 h, 550 °C for 4 h and 675 °C for 6 h [29]. The results showed improvements of both structural and mechanical characteristics. WAAM was used also to obtain samples of Cu-9Al-4Ni-4Fe-1Mn bronze, the mechanical and corrosion characteristics of which were determined by κ-phase precipitates [30].

The objective of this work was to study the effect of heat input, annealing, and pre-deformation/annealing on grain structures and mechanical characteristics of an EBAM grown thin-walled Cu-3wt.%Si-Mn bronze.

## 2. Materials and Methods

**Figure 1:**
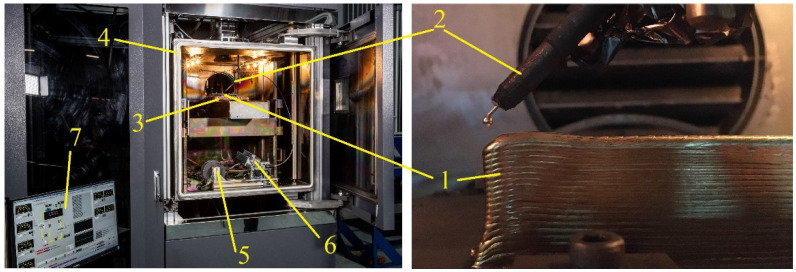
Electron beam wire-feed additive machine. 1—as-deposited wall, 2—wire guide, 3—water-cooled table, 4—vacuum chamber, 5—wire reel, 6—wire feeder, 7—control panel.

Thin-walled samples were manufactured using EBAM from Cu-3wt.%Si-0.8wt.%Mn bronze *∅*1.2 mm wire on a stainless steel substrate mounted on a water-cooled table inside the vacuum chamber of an electron beam wire-feed additive machine (Figure 1). Residual pressure in a chamber was ~8 × 10^−4^ Pa.

Different levels of heat input (Table 1) were used in EBAM. These heat input values were derived from previous experimenting and our earlier works [22,23]. The pre-deformation rate was evaluated experimentally for aluminum Cu-Al7 and silicon bronzes by applying successively thickness reduction values as follows: 3%, 6%, 9%, 12%, and 15%. It was established that ~9% deformation was enough to observe noticeable structural changes only in aluminum bronze but not in the silicon bronze. Additional experiments were carried out with 10–15% reduction, and it turned out that 10% was optimal while deformations at 12 and 15% were odd ones.

**Figure 2:**
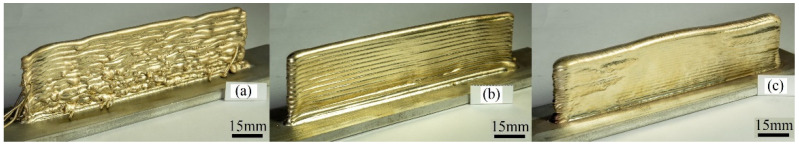
Optical images of thin walled samples obtained at heat input levels 0.19 kJ/mm (**a**), 0.21 kJ/mm (**b**), and 0.31 kJ/mm (**c**) (Table 1).

Using 0.19–0.31 higher heat input values resulted in lack of fusion between the successively deposited layers and severe geometry distortions, respectively (Figure 2a,c), which made it impossible to cut samples for mechanical testing. The best quality wall was obtained when using 0.21 kJ/mm heat input (Figure 2b).

**Figure 3:**
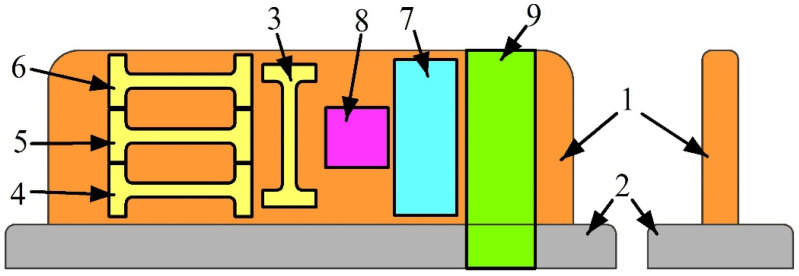
Sample cut-off scheme. 1—wall; 2—substrate; 3—sample with tensile axis orientation along the wall height; 4–6—samples with tensile axis orientation perpendicular to the wall height (along the layer deposition direction); 7—sample for post-treatment; 8—sample for XRD; 9—metallographic view sample.

Samples for microstructure examination and phase detection (Figure 3, pos. 8, 9), mechanical characteristics such as microhardness (Figure 3, pos. 9), and tensile strength (Figure 3, pos. 3–6) were cut off the as-built walls.

**Figure 4:**
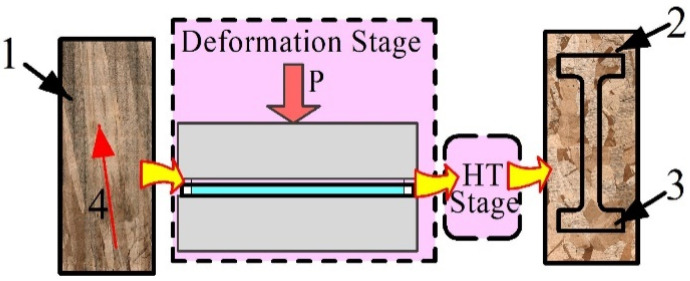
Scheme of pre-deformation and annealing of sample obtained at 0.31 kJ/mm. 1—sample before thickness reduction, 2—annealed after pre-deformation sample, 3—tensile test sample, 4—columnar grains. P—pressure, HT—heat treatment.

Post-treatment approaches such as high-temperature annealing of both as-deposited samples and pre-deformed samples were applied to refine their columnar grains and improve the mechanical characteristics. Samples intended for post-treatment were cut of the wall grown with heat input 0.31 kJ/mm, annealed at 900 °C for 6 h in a muffle furnace SNOL 7.2/1300 (SNOL, Utena, Lithuania), and then quenched in water (Figure 3, pos. 7). Another number of samples were pre-deformed by compression with thickness reduction 10% and then annealed according to the above-described procedure (Figure 4). It was mentioned above that parameters of such a treatment were determined from our earlier experience [22,23] using data of the Cu-Si diagram [31,32] and thermodynamic analysis [33,34].

Tensile tests were carried out using a tensile machine Testsystems 110M-10 (Testsystems, Ivanovo, Russia). The fracture surfaces were examined using a scanning electron microscope (SEM) Microtrac SemTrac mini (Microtrac Inc., Montgomeryville, PA, USA).

Metallographic polished views were prepared by grinding the cut-off samples on a grit 400–4000 emery paper and then polishing them on a 1 μm diamond paste. The polished surfaces were etched in a solution of composition as follows: 6 g FeCl_3_·H_2_O *+* 30 mL HCL *+* 60 mL of distilled water. After the etching the samples were washed in distilled water and dried, the microstructural characterization was performed with the use of a laser confocal microscope Olympus LEXT 4100 (Olympus Corporation, Tokyo, Japan).

Shimadzu XRD-7000S X-ray diffractometer (Shimadzu, Kyoto, Japan) was used to detect phases formed in the samples. High-resolution field emission scanning electron microscope (HR FESEM) Apreo 2 S (Thermo Fisher Scientific, Waltham, MA, USA), equipped with Velocity Super (EDAX, Mahwah, NJ, USA) with EBSD detector was used for obtaining grain orientation maps. The EBSD mode parameters were: 20 kV accelerating voltage and 25 nA probe current.

Specimens for EBSD were prepared by mechanical grinding on sandpaper from #400 to #2000 grit, diamond and colloidal silica polishing. Final step was ion polishing at 10 kV for 15 min using SemPrep 2 ion mill (Technoorg Linda, Budapest, Hungary).

## 3. Results

### 3.1. Metallography

**Figure 5:**
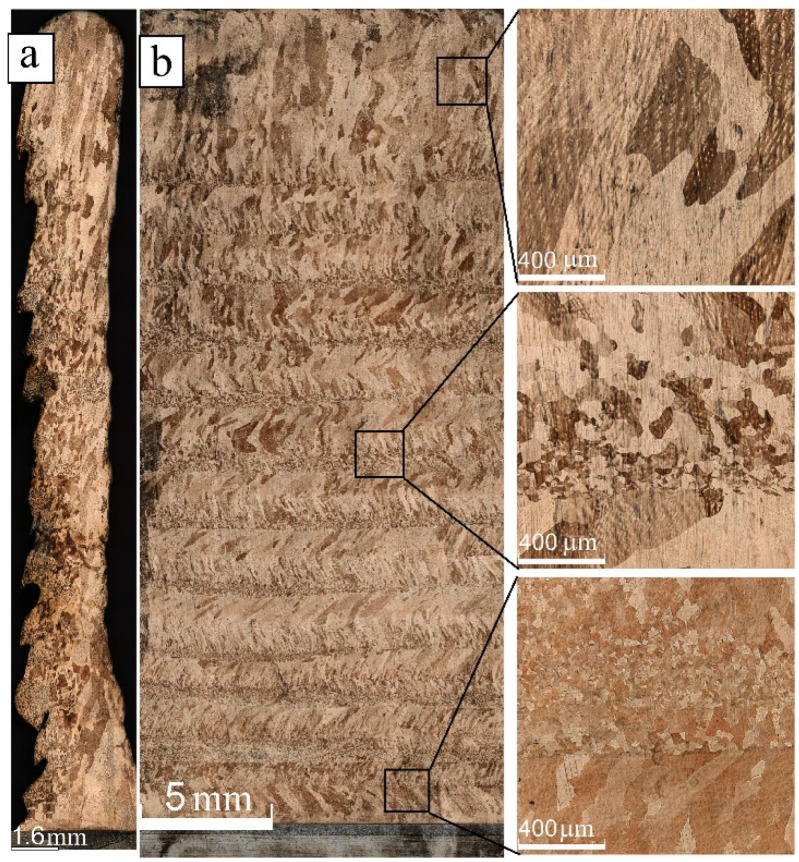
Profile (**a**) and front cross section optical view (**b**) of Cu-3wt.%Si-Mn bronze wall obtained at heat input of 0.19 kJ/mm with enlarged insets representing the bottom, medium, and top parts of the wall.

**Figure 6:**
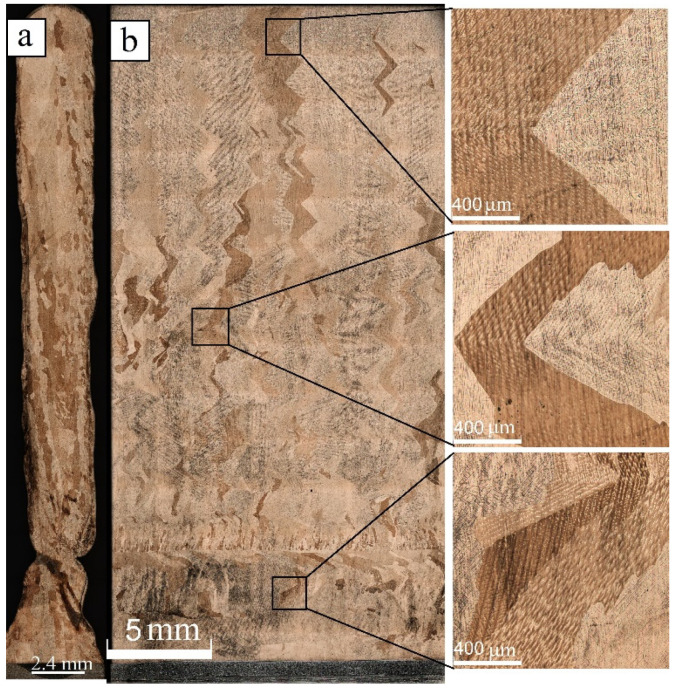
Profile (**a**) and front (**b**) cross section optical views of the Cu-3wt.%Si-Mn bronze wall obtained at heat input of 0.25 kJ/mm with enlarged insets representing the bottom, medium, and top parts of the wall.

**Figure 7:**
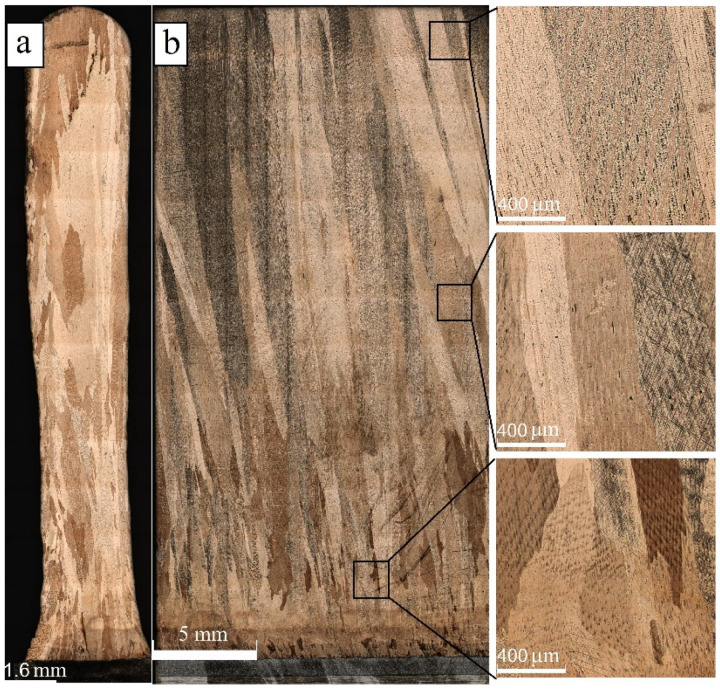
Profile (**a**) and front (**b**) cross section optical views of Cu-3wt.%Si-Mn bronze wall obtained at heat input of 0.31 kJ/mm with enlarged insets representing the bottom, medium, and top parts of the wall.

Optical metallography allowed evaluation of the heat input effect on the macrostructure of both as-deposited and post-treated silicon bronze samples. The as-deposited samples demonstrated grain structure differences depending upon the heat input values used during the additive manufacturing (Figure 5, Figure 6 and Figure 7). A profile view of the wall deposited at 0.19 kJ/mm in Figure 5a allows the observation of its non-uniform thickness as well as cold laps. A layer-by-layer nonhomogeneous macrostructure with alternating fine equiaxed and low aspect ratio columnar grains can be clearly seen in Figure 5b. The fine-grained interlayers are more clearly expressed in the bottom part of the wall where heat removal to the cold substrate was more effective as compared to that in the top part of the wall.

Additive deposition at medium heat input 0.25 kJ/mm resulted in forming a wall with smooth sides and some defects in the bottom part close to the substrate (Figure 6a). Columnar zig-zagged grain structures (Figure 6b) were formed in this sample. The layer-by-layer macrostructures are less expressed as compared to those formed at 0.19 kJ/mm. Only a singular small grain layer can be seen in the bottom part of the wall, where the heat removal rate was high enough.

Applying the maximum heat input of 0.31 kJ/mm allowed the growing of a uniform profile wall with smooth sides (Figure 7a). The typical as-cast microstructures with only high aspect ratio columnar grains formed there (Figure 7b). No zig-zagged or equiaxed smallgrains can be observed in this sample. This type of columnar grain microstructure is usual for the additive manufacturing and has a detrimental effect on mechanical characteristics of as-deposited metals, especially as soon as the copper alloys are concerned [1].

To sum up, three types of microstructures were obtained in response to varying the heat input, or to be more accurate, in response to the heat removal condition and solidification rate. Small equiaxed grains usually form at a high solidification rate while either dendrites or high aspect ratio columnar grains grow slowly from the melt. Some crossover regime can be achieved when premelting of the already-deposited layer is only superficial and part of melted wire bronze pool solidifies fast, thus forming these small grain layers. The upper portion of the pool solidifies slowly, thus giving the low-aspect ratio columnar grains.

**Figure 8:**
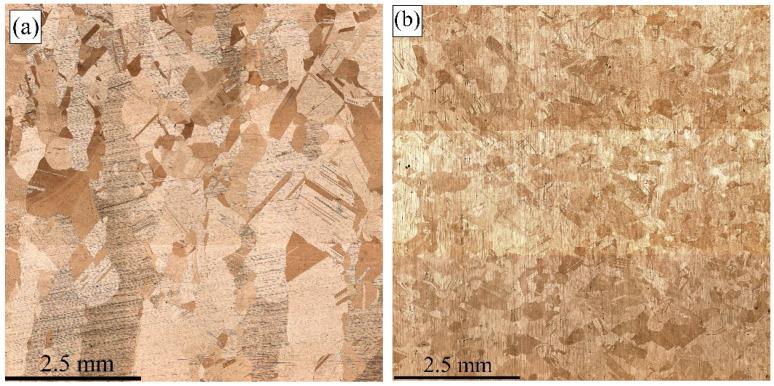
The microstructure of annealed at 900 °C for 6 h (**a**) and pre-deformed/annealed (**b**) sample of the Cu-3wt.%Si-Mn bronze deposited at 0.31 kJ/mm.

It was shown above that post-deposition treatment is a commonly accepted approach for mending such a coarse structure. Therefore, annealing of both as-deposited and pre-deformed 0.31 kJ/mm samples was carried out in an attempt to improve their characteristics. Annealing and quenching of the as-deposited 0.31 kJ/mm sample resulted in its essential structural modification by recrystallization (Figure 8a). The long columnar grains almost disappeared, thus giving a place to coarse irregular shaped ones transfixed with numerous annealing twins. Both coarse irregular shape grains and smaller equiaxed ones can be seen so that the microstructure can be characterized as nonhomogeneously sized grains, formed as a result of secondary recrystallization.

Annealing and subsequent quenching of the pre-deformed sample allowed the forming of new, finer recrystallized grains (Figure 8b). Pre-deformation at 10% served for introducing dislocations and deformation twins that facilitated recrystallization, and thus formed the microstructures as shown. Annealing twins also came into the picture, and it seems that they also became wider.

### 3.2. XRD

**Figure 9:**
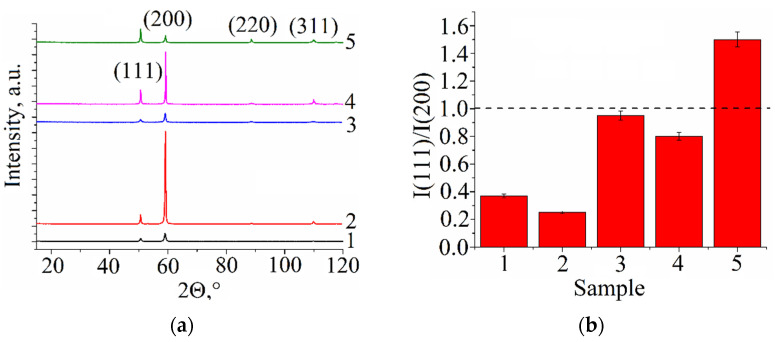
The XRD pattern (**a**) and texture intensity relationship (**b**) of Cu-3wt.%Si-Mn bronze samples: 1—heat input 0.19 kJ/mm, 2—heat input 0.25 kJ/mm, 3—heat input 0.31 kJ/mm, 4—as-annealed, 5—pre-deformed/annealed.

All as-deposited and post-treated samples were subjected to X-ray diffraction to detect and identify phases formed in them. It was established that all samples were composed of an FCC α-Cu phase that actually was a solid solution of the alloying elements in the Cu lattice. (Figure 9a). The inverse ratio of (111)_α_ and (200)_α_ peak intensities allows the suggestion of the existence of crystallographic textures (Figure 9b) in all as-deposited samples 1, 2, and 3, as well as in the as-annealed sample 4. Normal situation is observed only in the pre-deformed and annealed sample 5 where the (111)_α_ peak’s intensity is higher than that of (200)_α_, and the texture inherent in the as-deposited sample was destroyed by recrystallization.

### 3.3. Grain Orientation Maps

Using the EBSD method, it becomes possible to confirm or dispel the suspicion of the existence of crystallographic texture in our samples by reconstructing grain orientation maps (GOM). Studying the grain boundary misorientation distributions is another valuable method of microstructure examination.

**Figure 10:**
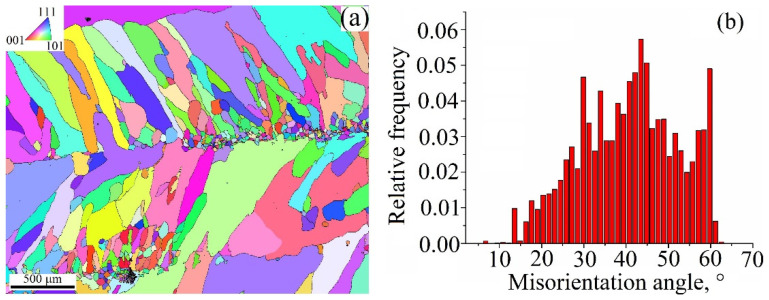
EBSD grain orientation map (**a**) and grain boundary misorientation angle distribution (**b**) in a cross section of the Cu-3wt.%Si-Mn bronze wall obtained at heat input of 0.19 kJ/mm.

The EBSD grain orientation map obtained from the 0.19 kJ/mm sample allows the observation of no preferential orientation of the grains, with approximately equal numbers of them oriented with their normals close to those found in the standard triangle corners (Figure 10a).

The thickness of the layer with low aspect ratio columnar grains is about 900 μm. The grains have both low and high angle boundaries, with a small percentage of special Σ3 ones belonging to annealing twins (Figure 10b). Grain boundary misorientation histograms in Figure 10b allow the quantitative observation of the changes that occurred in samples after deposition and post-treatments. An almost normal misorientation angle distribution of grain boundaries in the as-deposited 0.19 kJ/mm sample (Figure 10b) is slightly shifted to the high angle end.

**Figure 11:**
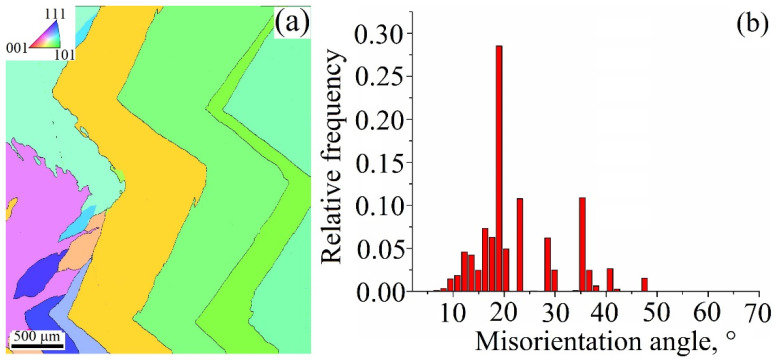
EBSD grain orientation map (**a**) and grain boundary misorientation angle distribution (**b**) in a cross section of the Cu-3wt.%Si-Mn bronze wall obtained at heat input of 0.25 kJ/mm.

The input sample deposited at 0.25 kJ/mm heat demonstrates its long zig-zagged grains with orientations close to [101] corner and [112] direction of the standard triangle (see the inset in Figure 11a). Judging by XRD, this sample is characterized by the highest number of grains oriented with respect to the [001] axis, i.e., has cubic growth texture value. However, EBSD results show the absence of [001] oriented grains in the same metallographic polished view. Such a discrepancy may be explained by the small number of grains visualized in the field of interest during EBSD as compared to that in XRD. In addition, the zig-zagged grains are not perfectly in line with the plane of view, in fact, they are inclined towards the back side of the wall. Such an inclination may interfere with the accuracy of the EBSD orientation detection. The biggest parts of the grain boundaries are the low angle ones (Figure 11b).

**Figure 12:**
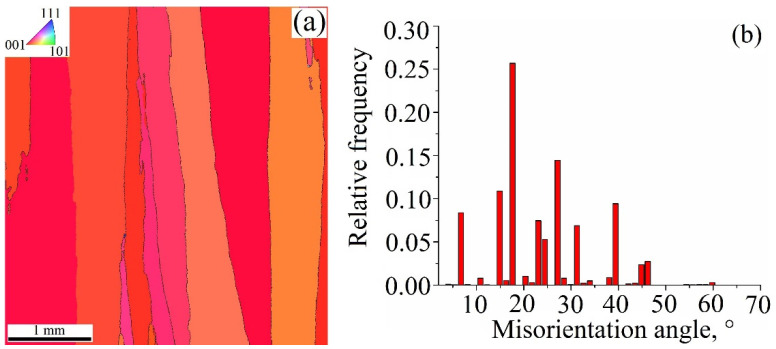
EBSD grain orientation map (**a**) and grain boundary misorientation angle distribution (**b**) in a cross section of the Cu-3wt.%Si-Mn bronze wall obtained at heat input of 0.31 kJ/mm.

Long columnar grains found in the 0.31 kJ/mm sample have their orientations close to those in the [001] corner of the standard triangle (Figure 12a). Such a finding can be confirmation by the cubic texturing. Grain boundaries are mostly the low angle ones (Figure 12b).

**Figure 13:**
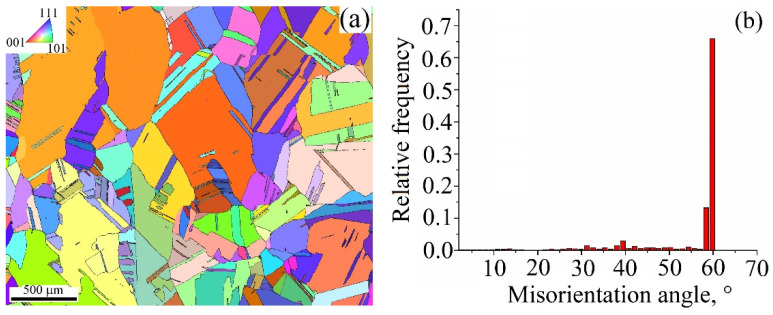
EBSD grain orientation map (**a**) and grain boundary misorientation angle distribution (**b**) in a cross section of annealed Cu-3wt.%Si-Mn bronze annealed at 900 °C for 6 h.

Annealing the 0.31 kJ/mm sample at 900 °C followed by quenching in water resulted in fixing the high-temperature state that can be characterized by destroying the cubic texture, forming the non-homogeneous grain structure and annealing twins (Figure 13a). Annealing the 0.31 kJ/mm sample gives almost 100% of high-angle boundaries including ~65% of twin ones (Figure 13b). Correspondingly, there are numerous Σ3 twin boundaries (Figure 13b).

**Figure 14:**
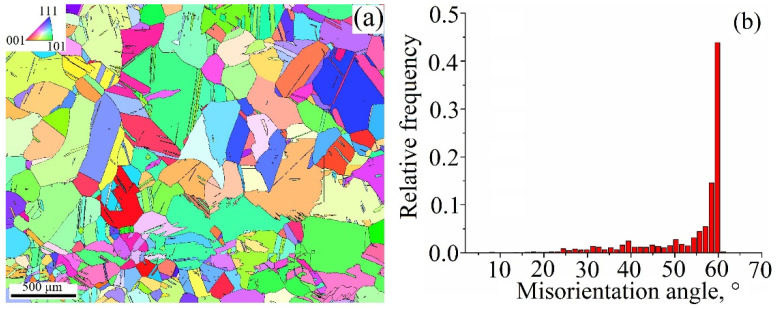
EBSD grain orientation map (**a**) and grain boundary misorientation angle distribution (**b**) in a cross section of Cu-3wt.%Si-Mn bronze 10% pre-deformed and then annealed at 900 °C for 6 h.

**Figure 15:**
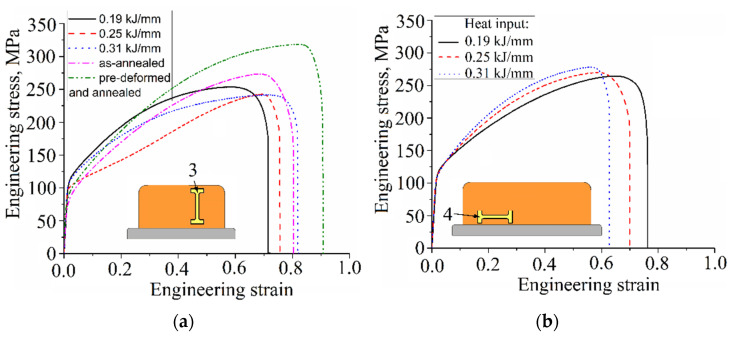

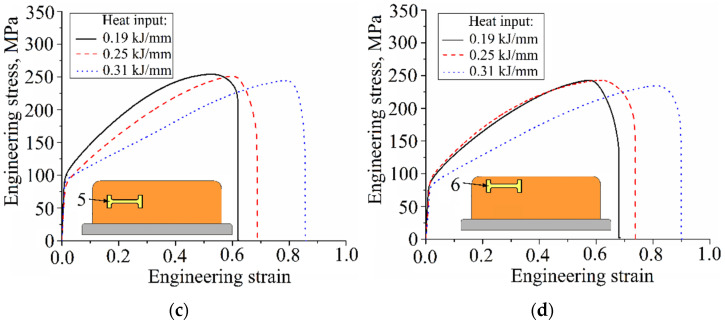
The “stress-strain” curves obtained from tensile tests on Cu-3wt.%Si-Mn bronze samples 3 (**a**), 4 (**b**), 5 (**c**) and 6 (**d**) cut off the different parts of the wall.

Annealing the 10% pre-deformed 0.31 kJ/mm sample also gives the non-homogeneous grain structures with annealing twins (Figure 14a). However, in this case, the grains look smaller. There are no enormous larger 1 mm grains that can be seen in Figure 13a. As suggested, plastic deformation served to recrystallize the grains during annealing so that some of them then grew to reach the size of ~500 μm. Many equiaxed grains appeared there as a result of primary recrystallization. Annealing of the pre-deformed 0.31 kJ/mm sample gives ~45% of twin boundaries but higher percentage of the boundaries with misorientation angles in the range 25 to 59° (Figure 15b).

### 3.4. Mechanical Characteristics

Tensile test samples were cut off different as-deposited wall parts to help analyze the effect of grain structure nonhomogeneity on their mechanical characteristics (Figure 3, pos. 4, 5, 6). This, however, was not fulfilled in case of testing the post-treated samples when samples were cut off so that their tensile axes were oriented along the wall height (Figure 3 and Figure 4, pos. 3).

Stress/strain curves in Figure 15a characterize the tensile loading behaviors of as-deposited samples cut off the walls according to Figure 3, pos. 3, with their tensile axes oriented along the wall height. All the as-deposited samples demonstrated rather high levels of ductile properties so that strain-to-fracture values were in the range 60–90%. The magnitudes of both yield stress (YS) and ultimate tensile strength (UTS) vary in the ranges 78–109 MPa and 234–250 MPa, respectively, with the maximum value inherent with the 0.19 kJ/mm sample. Two other samples obtained at 0.25 and 0.31 kJ/mm demonstrated lower UTS but higher strain-to-fracture values. These results can be explained by microstructural differences among all three samples.

Specific zig-zagged grain structures of the 0.25 kJ/mm sample manifested in the specific shape of corresponding stress/strain curve in Figure 15a, with easy plastic deformation stage.

The annealed sample demonstrated the plasticity characteristic close to those of 0.31 kJ/mm sample but with higher UTS values. The pre-deformed and annealed sample showed simultaneously improved strength and plasticity.

The as-deposited tensile samples 4, 5, and 6 sampled from the bottom, medium, and top parts of the wall, respectively (Figure 15a–c), were oriented by their tensile axes across the wall’s height, i.e., along the layer deposition direction. Corresponding stress/strain curves revealed decreasing UTS and increasing strain-to-fracture as the heat input increased. The difference between the curves in Figure 15b is minor as compared to those in Figure 15c, and especially in Figure 15d.

**Figure 16:**
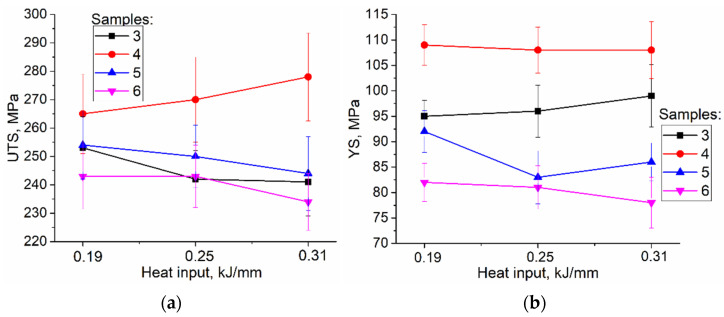
UTS (**a**) and YS (**b**) vs. heat input dependencies for tensile samples cut off different wall parts. 3 to 6 relate to testing samples representing the different parts the wall (see Figure 3).

The UTS values increase with the heat input only for sample 4 that was cut from the bottom parts of the walls, while the UTS values of samples 5 and 6 reduce as the heat input increases (Figure 16a); the latter is true also for sample 3 with its tensile axis oriented along the wall’s height. The YS values of sample 4 are higher than those of samples 5 and 6 (Figure 16b). Sample 5 demonstrates minimal YS value for heat input 0.25 kJ/mm when the zig-zagged structure is formed.

**Figure 17:**
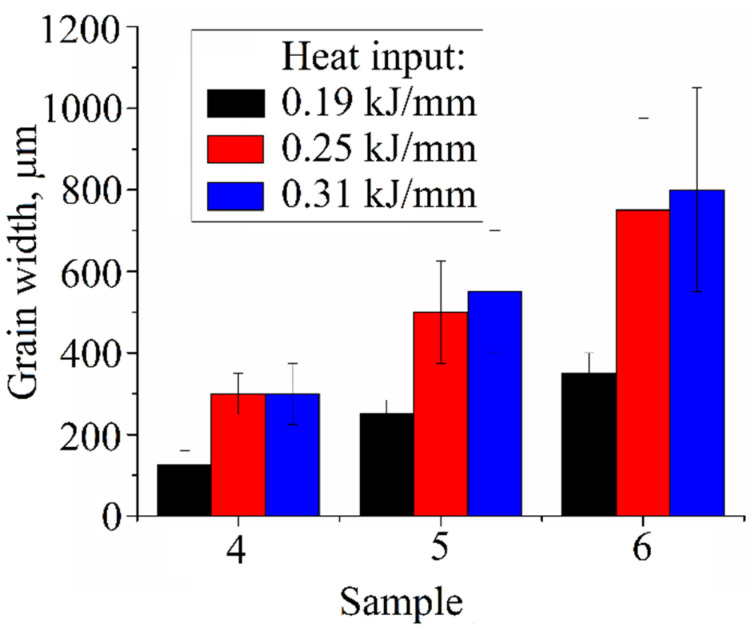
Grain width vs. heat input dependencies for samples 4, 5, and 6.

In general, there is an inverse correlation between UTS and columnar grain width (Figure 17), i.e., UTS (Figure 16a) is reduced when columnar grains become wider at higher heat input. Similar results were reported for additively manufactured Cu-7.5 wt.%Al [23]. However, the UTS values of the as-deposited sample 4–cut off the bottom part of the walls–show a tendency to increase the UTS with the heat input. Such a tendency can be explained by the existence of a transition bronze/steel zone, which becomes more saturated with steel at higher heat input.

**Figure 18:**
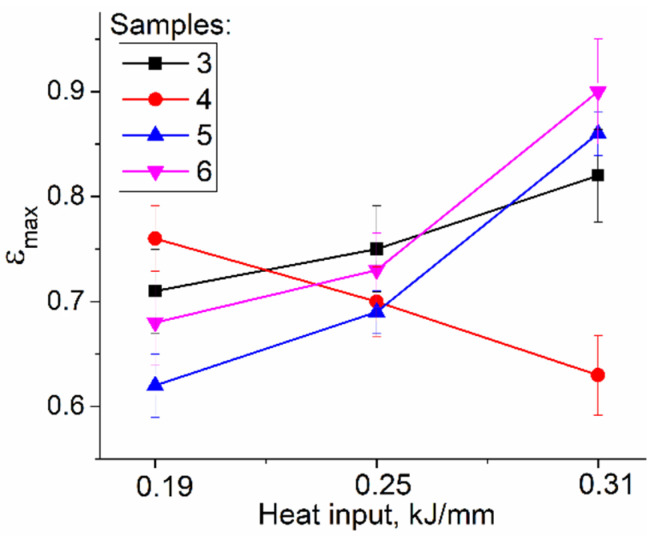
Strain-to-fracture vs. heat input dependencies for tensile samples cut off different Cu-3wt.%Si-Mn wall parts. 3 to 6 relate to testing samples representing the different parts the wall (see Figure 3).

The effect of heat input on ductility characteristics of the samples is more pronounced. Samples 5 and 6 that represent the medium and top parts of the walls, respectively, show an increasing of strain-to-fracture with the heat input values (Figure 18). The same is true for sample 3, the tensile axis of which is parallel to the wall’s height. Sample 4–cut off the bottom part of the wall–revealed an inverse behavior when strain-to-fracture decreased as the heat input increased. All samples demonstrated minimal strain-to-fracture value scatter in samples deposited at 0.25 kJ/mm.

**Figure 19:**
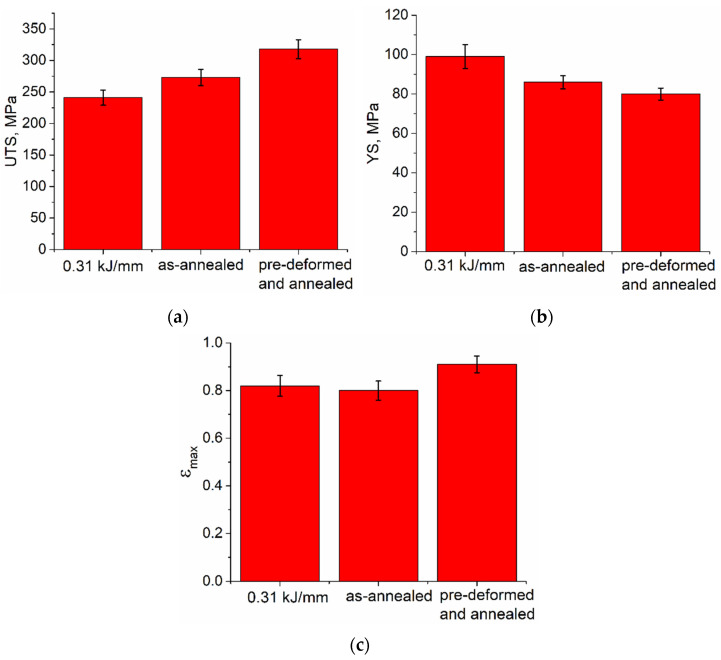
UTS (**a**), YS (**b**) and strain-to-fracture (**c**) characteristics of the 0.31 kJ/mm as-deposited, annealed and pre-deformed/annealed Cu-3wt.%Si-Mn samples.

Annealing of the 0.31 kJ/mm sample resulted in increasing the UTS by 13% (Figure 19a) and decreasing YS and strain-to-fracture by 13% (Figure 19b) and 2.5%, respectively (Figure 19c). Pre-deformation and annealing increased the UTS (Figure 19a) and strain-to-fracture (Figure 19c) by 35% and 20%, respectively, as well as decreased the YS by 20% and 2.5% (Figure 19b).

The microstructures formed in the samples after the post-treatments provided improved strength characteristics by eliminating the as-deposited columnar grains and creating new high angle grain boundaries, including the twin boundaries, which are very effective barriers against dislocation gliding.

**Figure 20:**
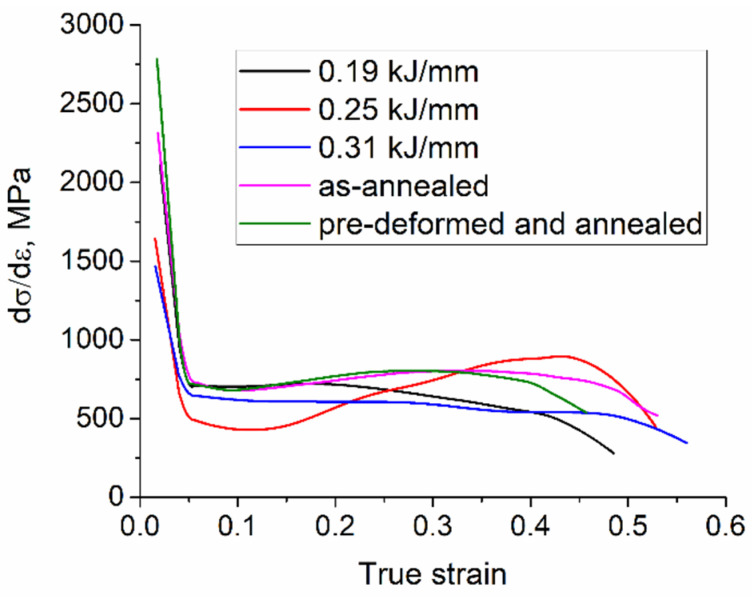
Strengthening rate vs. strain dependence for tensile tests of Cu-3wt.%Si-Mn bronze samples with tensile axis along the wall’s height (see position 3 in Figure 3 and diagrams in Figure 16a).

It is known [8] that plastic deformation mechanisms in silicon bronze occur by dislocation movement and twinning. The behavior of samples and contributions of different mechanisms of plastic deformation can be analyzed using a dependence of strengthening rate dσdε on the strain (Figure 20). The first decaying portions of the curves correspond to the dislocation movement in the grains; the easiest dislocation gliding occurs in the 0.25 kJ/mm sample (red line). It is suggested, then, that starting from ε = 0.15 ÷ 0.2, all curves except that of 0.31 kJ/mm reveal strengthening rate growth due to deformation twinning.

High ductility of the 0.31 kJ/mm sample can be the effect of forming the high aspect ratio columnar grains, which can easily elongate during tensile testing. Such an effect was reported earlier [23]. Therefore, dislocation gliding is the main deformation mechanism with this sample.

The 0.25 kJ/mm sample shows some specifics in tensile behavior due to its zig-zagged columnar grains. These grains easily elongate at low strain values but, starting from ε = 0.15, a rising portion of the curve that can be referred to as the twinning stage was observed. This stage lasts up to the ε = 0.3 point.

Nonhomogeneous microstructures of the 0.19 kJ/mm sample combine low aspect ratio columnar grains with interlayers of equiaxed smaller ones. In fact, dislocation gliding becomes easy in the columnar grains, while small grain interlayers with many grain boundaries offer more dislocation barriers.

Annealing of the 0.31 kJ/mm sample resulted in structurally non-homogeneous structures with new coarse grains and twin boundaries. The twinning stage starts from ε = 0.15, reaches its maximum at ε = 0.3, and then decays. The same behavior could be observed in case of tensile test of the pre-deformed and annealed sample, except for a faster decay stage.

### 3.5. Fractography

**Figure 21:**
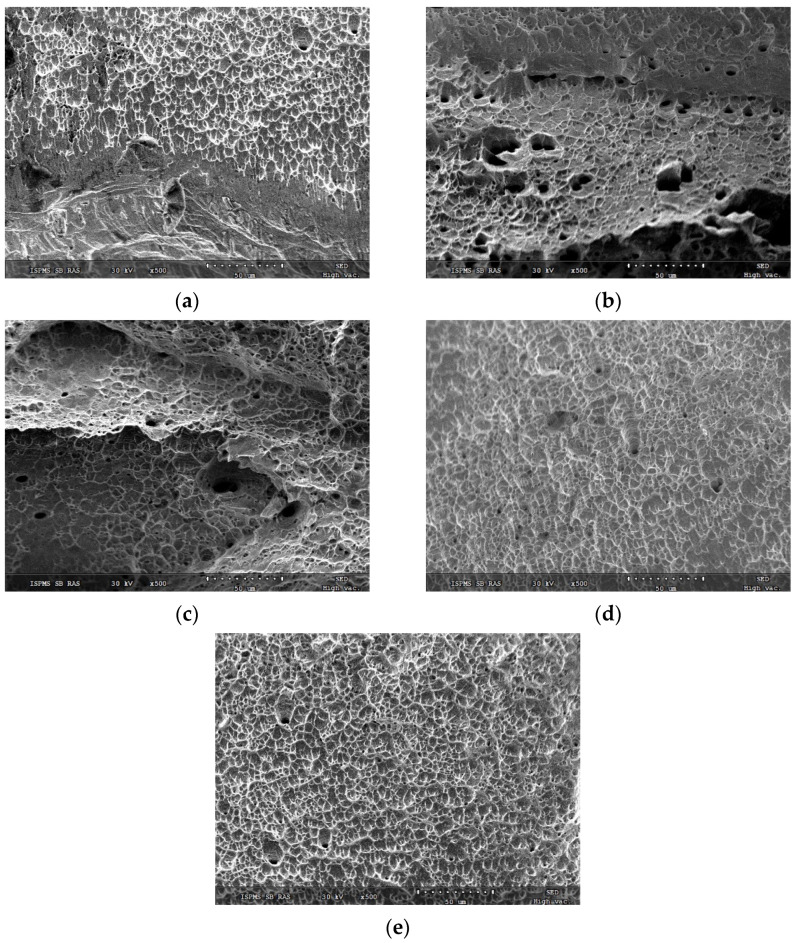
Fracture surface after tension test for Cu-3wt.%Si-Mn bronze samples (Figure 3, pos. 3) printed at 0.19 kJ/mm (**a**), 0.25 kJ/mm (**b**) and 0.31 kJ/mm (**c**) heat input, annealed (**d**), pre-deformed/annealed (**e**).

Fracture surfaces of all as-deposited and post-treated samples were examined for dominating fracture mechanism. Corresponding SEM SE images demonstrated that all fracture surfaces resulted from a quasi-viscous fracture (Figure 21). The fracture surface of the 0.19 kJ/mm sample (Figure 21a) revealed relatively smooth areas in addition to viscous mode dimples, while both 0.25 (Figure 21b) and 0.31 kJ/mm samples (Figure 21c) were characterized by coarse ridges that could appear from the intercrystalline fracture. Both annealed (Figure 21d) and pre-deformed (Figure 21e) 0.31 kJ/mm samples demonstrated pure viscous fracture surfaces without any coarse ridges or smooth areas.

### 3.6. Microhardness

**Figure 22:**
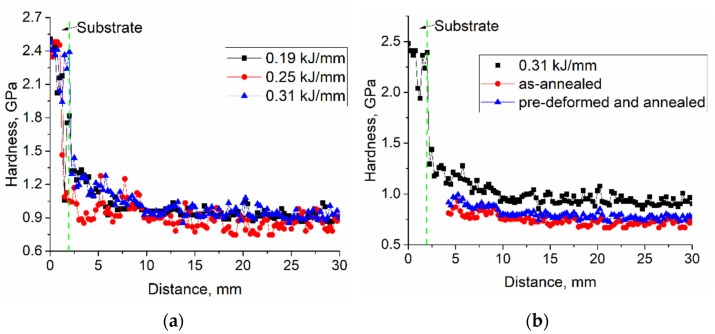
Microhardness profiles obtained along the walls height for as-deposited (**a**) and post-treated (**b**) Cu-3wt.%Si-Mn bronze samples.

Microhardness profiles obtained along the wall’s height on as-deposited samples demonstrated a steel/bronze transition zone in the bottom part of the wall where melted steel substrate intermixed with the deposited bronze (Figure 22a). Since copper and iron are immiscible metals, intermixing provides enhanced hardness due to the presence of steel grains. It is also possible that some nickel was dissolved in the bronze grains and thus somewhat contributed to hardening [14]. The as-deposited bronze microhardness is at the level of ~0.8–1.1 GPa.

The microhardness numbers of the annealed and pre-deformed 0.31 kJ/mm samples are ~0.73 GPa and ~0.8 GPa (Figure 22b), i.e., somewhat lower than ~0.96 GPa of the parent as-deposited samples.

## 4. Discussion

This work is part of research focused on finding methods for improving microstructural and mechanical characteristics of additively manufactured metals and alloys. Cu-3wt.%S-0.8wt.%Mn bronze is a single phase FCC alloy with low stacking fault energy, the plastic deformation of which occurs by dislocation gliding and twinning [8]. It is structurally similar to the earlier reported additively manufactured Cu-7.5wt.%Al bronze [23], and therefore one of the objectives was to compare between types of microstructures obtained at different heat input levels.

The above results showed that microstructures formed in the additively manufactured Cu-3wt.%S-0.8wt.%Mn depended on the heat input level. The insufficient heat input resulted in forming cold laps on the wall sides, uneven thickness, poor fusion between the successively deposited layers, etc. The small grain structures were the result of fast solidification of insufficiently heated pool by fast heat removal across the fusion boundary. Such a type of structure is often observed in the vicinity of the cold substrate and disappears with an increase in the number of layers deposited and/or heat input. This type of microstructure could be desirable if spread over the total wall’s volume. However, there were columnar type grains with an aspect ratio that depended on the heat removal conditions too.

Medium heat input resulted in forming the zig-zagged long grains according to mechanisms reported elsewhere [35]. Let us note that the same type of structure was formed in Cu-7.5wt.%Al bronze at heat input of 0.225 kJ/mm [23]. It seems that this type of microstructure is a crossover from the fast-cooled equiaxed grained type to a fully high aspect ratio columnar. Tensile tests show that the zig-zagged grains attain less plasticity to the samples as compared to that of pure columnar ones. Such a behavior was also observed in tensile tests of the Cu-7.5wt.%Al bronze samples [23]. However, crystallographic orientation of these grains with respect to tensile axis will play more important role. The zig-zagged shape of such a grain may have its effect on deformation and reorientation of its differently directed parts. Furthermore, these grains clearly demonstrated the twinning stage starting from ε = 0.15, i.e., later than that of 0.19 kJ/mm samples (Figure 21).

Fully columnar high aspect ratio grains obtained at 0.31 kJ/mm demonstrated higher ductility and a slightly lower strength as compared to the zig-zagged ones obtained at 0.25 kJ/mm. No twinning stage was observed for these samples (Figure 21).

A tendency for reduction of UTS with an increase in heat input and, correspondingly, the grain size, can be explained by taking into account the Hall-Petch law. The larger is the grain, the longer the dislocation free run path, and the less plausible twinning is. Therefore, high aspect ratio columnar grains were deformed only by the dislocation gliding mechanism without initiation of any twinning stage.

Annealing of the 0.31 kJ/mm samples resulted in recrystallization and grain growth of the recrystallized grains according to the secondary recrystallization mechanism. Numerous annealing twins are the result of primary recrystallization, and can serve as barriers against dislocations. It seems that no primary high aspect ratio grains survived the annealing at 900 °C for 6 h, judging by the fact that none of them (Figure 13a) demonstrate their long axis orientation coinciding with [001]-axis (Figure 12a). Large ~1 mm sized grains that have grown according to secondary recrystallization mechanism can be seen in Figure 13. The presence of such a grain size nonhomogeneity is the result of exposing the samples to high temperatures for a long time. Using lower temperatures and less annealing time would allow the obtaining of more uniform grain size distribution. Nevertheless, even such non-optimal annealing conditions allowed the strength improvement of the as-deposited samples. All the above can be related to the pre-deformed and annealed samples that had a strength that was not only higher than that of just the annealed sample, but where simultaneous improvement of ductility and strength was also achieved.

## 5. Conclusions

The effects of both heat input during additive manufacturing and post-processing treatments on structural evolution and mechanical characteristics of the electron beam additive manufactured Cu-3wt.%Si-Mn thin walls have been investigated.

Increasing the heat input in electron beam additive manufacturing of Cu-3wt.%Si-0.8 wt.%Mn on a cooled stainless steel substrate resulted in microstructural modification of wall samples from a bimodal equiaxed/low aspect columnar grain structure to zig-zagged, and then to fully high aspect ratio columnar.

Annealing at 900 °C for 6 h resulted in elimination of the high-aspect ratio columnar grains and forming non-homogeneous grain structure and annealing twins. The annealed sample’s ultimate tensile strength increased by 13%, while yield stress and strain-to-fracture decreased by 13% and 2.5%, respectively, as compared to those of the as-deposited at 0.31 kJ/mm sample.

The use of pre-deformation and subsequent annealing allowed completely destroy the columnar grain structure to the advantage of obtaining a polycrystalline, texture-less equiaxed one. Simultaneous improvement of strength by 20% and ductility by 11% has been achieved, as compared to those of the as-deposited at 0.31 kJ/mm sample. Therefore, the post-processing that combined deformation and annealing proved to be more effective, as compared to annealing.

The above-disclosed results can be used for developing new methods to control structure formation during electron beam additive manufacturing. However, the use of pre-deformation/annealing treatment on shaped or large-scale components is rarely easy. Therefore, further development in electron beam additive manufacturing of bronzes can be associated with the use of in-situ interlayer deformation methods.

## Figures and Tables

**Table 1:** EBAM process parameters.

Regime	Beam Current, mA	Layer Deposition Rate, mm/min	Accelerating Voltage, kV	Heat Input, kJ/mm
1	25	240	30	0.19
2	33	240	30	0.25
3	41	240	30	0.31

## Data Availability

Data will be made available based on request to the authors.
